# Enhancing Skin Cancer Detection and Classification in Dermoscopic Images through Concatenated MobileNetV2 and Xception Models

**DOI:** 10.3390/bioengineering10080979

**Published:** 2023-08-19

**Authors:** Roseline Oluwaseun Ogundokun, Aiman Li, Ronke Seyi Babatunde, Chinecherem Umezuruike, Peter O. Sadiku, AbdulRahman Tosho Abdulahi, Akinbowale Nathaniel Babatunde

**Affiliations:** 1 Department of Computer Science, Landmark University, Omu Aran 251103, Nigeria; 2 Department of Multimedia Engineering, Kaunas University of Technology, 44249 Kaunas, Lithuania; 3 School of Marxism, Guangzhou University of Chinese Medicine, Guangzhou 510006, China; 4 Department of Computer Science, Kwara State University, Malete 241103, Nigeria; 5 Department of Software Engineering, Bowen University, Iwo 232102, Nigeria; 6 Department of Computer Science, University of Ilorin, Ilorin 240003, Nigeria; 7 Department of Computer Science, Kwara State Polytechnic, Ilorin 240211, Nigeria

**Keywords:** skin cancer, deep convolutional neural network, transfer learning, data augmentation, deep learning

## Abstract

One of the most promising research initiatives in the healthcare field is focused on the rising incidence of skin cancer worldwide and improving early discovery methods for the disease. The most significant factor in the fatalities caused by skin cancer is the late identification of the disease. The likelihood of human survival may be significantly improved by performing an early diagnosis followed by appropriate therapy. It is not a simple process to extract the elements from the photographs of the tumors that may be used for the prospective identification of skin cancer. Several deep learning models are widely used to extract efficient features for a skin cancer diagnosis; nevertheless, the literature demonstrates that there is still room for additional improvements in various performance metrics. This study proposes a hybrid deep convolutional neural network architecture for identifying skin cancer by adding two main heuristics. These include Xception and MobileNetV2 models. Data augmentation was introduced to balance the dataset, and the transfer learning technique was utilized to resolve the challenges of the absence of labeled datasets. It has been detected that the suggested method of employing Xception in conjunction with MobileNetV2 attains the most excellent performance, particularly concerning the dataset that was evaluated: specifically, it produced 97.56% accuracy, 97.00% area under the curve, 100% sensitivity, 93.33% precision, 96.55% F1 score, and 0.0370 false favorable rates. This research has implications for clinical practice and public health, offering a valuable tool for dermatologists and healthcare professionals in their fight against skin cancer.

## 1. Introduction

**Figure 1:**
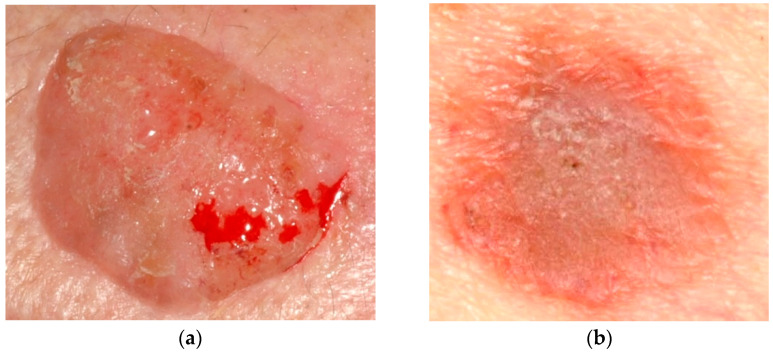
(**a**) Cancer image and (**b**) non-cancer image from the dataset.

Since the beginning of the 20th century [1], many people of all genders have been diagnosed with skin cancer (SC). In 2012 in the United States, there were about 76,250 newly diagnosed cases of melanoma and 8790 fresh fatalities attributed to melanoma [2]. It is estimated that there were 165,580 new instances of non-melanoma SC in Brazil during the biennium of 2018–2019 [3]. The progression of skin cancer is caused by several reasons, including the long-life expectancy of the population, skin exposure to the sun, and the early identification of SC. One of the most trustworthy early skin cancer identification procedures is dermoscopy, a noninvasive imaging technique performed on the skin. The appearance of skin lesions can alter dramatically from a dermoscopic picture to a dermoscopic image, depending on the state of the skin [4]. In addition, the presence of various artifact sources, such as hair, skin texture, or air bubbles, might increase the likelihood of incorrectly recognizing the border between the skin lesions and the healthy skin surrounding them. Even though dermoscopy is a competent method for identifying SC, it is very challenging for even the most experienced dermatologists to accurately categorize malignant and benign skin lacerations from several dermoscopy pictures. As a result, it is of the utmost need to create an effective computer-aided diagnostic (CAD) system that does not involve any invasive procedures for categorizing skin lesions [5,6]. Image preprocessing, feature extraction, segmentation, and categorization are the four primary stages of the CAD scheme. It is essential to remember that the categorization effectiveness of the entire CAD scheme is strongly impacted by each step [7,8,9]. As a result, it is necessary to use practical algorithms at each phase to accomplish excellent diagnostic performance. Figure 1 displays a portion of the picture that is included in the dataset as a sample. The photos are saved in JPG format and vary in the number of pixels they possess.

Several research studies [10,11,12,13] looked at various machine learning strategies to detect multiple forms of diseases such as cancer. Most of this research used classifiers with very simple architectures that were trained on a collection of manually produced characteristics taken from the photos [14,15]. Most algorithms for machine learning (ML) bring a significant amount of computing time for an accurate diagnosis, and their effectiveness is contingent on the characteristics chosen to define the malignant area. The utilization of DL and convolutional neural networks (CNNs) [16,17,18,19], both neural networks, has emerged as an effective method for the automated detection of many types of cancer [20]. The field of picture categorization, which includes the examination of skin lesions, has seen remarkable progress thanks to deep learning [21]. Transfer learning [22] and data augmentation [23] are methods used in picture classification tasks to compensate for a data shortage and lower computing and memory necessities.

Artificial intelligence (AI) is new; its revolution is analogous to the upheaval brought about by adding technologies to all aspects of our life [24,25,26]. ML techniques eliminate the time-consuming stage of manually extracting features and facilitate the quick completion of classification projects [27]. Recently, there has been an increasing interest in using ML techniques to identify cancer precisely [27,28]. Over the last several decades [28], advancements in ML procedures have enhanced cancer identification accuracy by 15 and 20 percent. Deep learning (DL) is one of the domains of artificial intelligence that is expanding at the fastest rate owing to the many fields in which it is used [29,30,31,32,33]. DL, and especially CNNs, driven by advanced computer algorithms and enormous datasets, has become one of the greatest effective and widespread ML methods in picture recognition and categorization [34]. It has also been utilized to identify skin lesions [35,36]. There is no longer a necessity for the preliminary information and intricate picture pretreatment procedures necessary for image classification when utilizing classic ML approaches. It has been established that some deep learning-based classifiers can classify skin cancer photos with the same level of accuracy as dermatologists [36]. Consequently, CNNs can potentially contribute to developing computer-aided fast skin lesion classifiers on par with dermatologists’ use.

Transfer learning (TL) has shown to be beneficial for dealing with relatively small datasets, such as medical photographs, which are more difficult to gather in large quantities than other datasets. It is frequently more practical to employ a model that has already been trained and fine-tune its efficacy to shorten and speed up the procedure. Preparing a NN from scratch would necessitate actual data and a lot of processing effort. On the ImageNet Large Scale Visual Recognition Challenge (ILSVRC) dataset, many already pre-trained CNNs were trained. These CNNs include AlexNet, Inception, ResNet, and DenseNet [37,38]. Several CNNs that had been pre-trained accomplished good categorization efficacy in detecting skin cancer [39].

The term “data augmentation” refers to a technique that generates additional data based on the original data that are being supplied [39]. This helps to increase the amount of data that is being input. Image enhancement techniques may be used to make up for the shortcomings of the dataset about skin cancer. Data augmentation might be proficient in a diversity of ways, such as by rotating the data, scaling it, cropping it at random, or modifying its color. Data augmentation is used extensively when pre-trained CNN architectures are used. In [40], the effectiveness of skin lesion categorization is improved by applying data augmentation (DA) with geometric transformations (rotations by multiples of 90 degrees and lesion-preserving cropping). This is accomplished by improving the classification accuracy [41]. In [39], the impact of DA was examined across several binary classifiers trained using features retrieved by a pre-trained Inception-v4 system. Dermoscopic pictures were used in the study referenced in [29] to classify melanoma, seborrheic keratosis, and nevocellular nevus utilizing a deep learning technique. A deep CNN prediction technique built on a novel regularized approach was used in [42] to categorize skin lesions, achieving an accuracy of 97.49%.

For the last ten years, many novel and, generally speaking, more effective methods for treating cancer have evolved [43,44,45,46]. These methods include immunotherapies and targeted medicines. Researchers are presently focusing on creating medications that directly target particular mutations in melanoma cells or employ the body’s immune system to combat melanoma [47]. Thanks to contemporary epigenetics [48], these efforts are already underway. It is thought that modifying one’s behavior, being vaccinated, and taking certain drugs may prevent more than half of all malignancies. According to the findings of the study [49], it is possible that a significant number of cases of skin cancer may be avoided. Examining the skin for abnormal changes at an early stage may assist in diagnosing skin cancer and prevent the disease from spreading to other organs. It is essential to the patient’s health that malignant tumors be diagnosed and graded as early as possible. In cases when melanoma is identified too late, the disease may have already spread to deeper layers of the skin. Treatment options become increasingly limited when the condition progresses to a later stage. Dermatoscopy is now the method of choice for early detection, even though it is a challenging technique for seasoned dermatologists. Patients are at an additional disadvantage because they need to make numerous visits to the doctor to monitor and spot changes in their skin color. This therapy takes a very long time, and there is a high risk of making mistakes, which puts the patient’s life in danger. Because of this, technology is needed to identify and categorize skin cancer cases more quickly and accurately [50].

Many different strategies have been published in the research literature [50,51,52,53] that can detect cancer from relatively few datasets. However, the influence this will have on a vast database has not been well researched. There are several categorization algorithms, most of which depend primarily on manually produced feature sets. However, these feature sets have limited ability for generalization in dermatoscopic skin pictures. Lesions exhibit a significant visual resemblance to one another because of their closeness in color, shape, and size; consequently, lesions are highly connected, resulting in inadequate feature information. As a direct consequence, feature-based skin categorization methods developed by hand are useless [50,51,52,53,54]. A strategy that is based on deep learning (DL) is helpful in this specific scenario. Deep learning (DL) systems have a substantial capacity to extract complex, detailed, task-specific, and practical features, which enables them to produce an elegant model with enhanced performance without the hand-crafted features needed by standard machine learning (ML) techniques [55,56]. This ability allows DL systems to create a luxury model with improved performance. The detection of skin cancer with DL is efficient in terms of cost, and the need for dermatoscopic examination may be reduced. As a result, illnesses that might lead to skin cancer can be recognized in their earlier stages using the approach described in this study.

This study demonstrates a novel, very effective, and precise DL model for the quick and painless diagnosis of skin cancer using dermatoscopy in patients, including those who may have a pathogenic infection. With a prediction accuracy of 97.56%, the proposed hybrid concatenated SkinNetX model classifies the skin cancer picture dataset into two different forms of cancer. The dataset contains photos of skin cancer. To speed up the screening process for skin cancer, the suggested method might be combined with a dermatoscopic examination. Therefore, the presented technique might allow us to circumvent the delay in dermatoscopy. As a result, it can serve as an alternate technique for speedy skin cancer diagnosis. In addition, automated categorization of lesions can assist medical professionals in their day-to-day clinical practice and enable prompt and cost-effective access to potentially life-saving diagnoses, even outside of the hospital setting.

The primary focus of this study is building a novel skin cancer detection and classification system that leverages the strengths of MobileNetV2 and Xception algorithms. By concatenating these two CNN architectures, we aim to achieve improved accuracy, robustness, and generalization capabilities in identifying skin cancer. The main contributions are as follows:

- Improved accuracy and robustness in skin cancer detection and classification
- Enhanced generalization capabilities, enabling accurate identification of skin cancer
- A comparative examination indicating the superiority of the suggested concatenated model over individual MobileNetV2 and Xception models, as well as other existing approaches
- Real-world application with a user-friendly interface for efficient and reliable skin cancer screening

The remaining parts of the manuscript are organized as described below. Section 2 discusses the existing works related to the present study that were considered. The section also discusses in detail the categorization of datasets as benign or malignant, which presents the approach that was applied in this particular research. The collected findings and the metric assessment are shown in Section 3, which is followed by Section 4 Results and Discussion. The primary observations and judgments are presented in Section 5. The study is also concluded here, and future work is suggested and recommended.

## 2. Related Works

Several researchers have conducted studies on detecting and diagnosing various diseases, such as Parkinson’s [57], hyperspectral anomaly [58], emotion detection [59], and diabetic retinopathy [60]. More and more individuals are being diagnosed with skin cancer due to the rapid increase in global air pollution and the reduction in the atmosphere’s ozone. As a direct consequence, researchers have been working to build computerized systems that can diagnose skin cancer based on pictures obtained from dermatoscopic examinations. The approaches that originated from computer vision are ML and DL. Below is a discussion of the many techniques that are offered in the relevant literature.

Most systems [61,62,63,64] extract characteristics from images using various image processing techniques and then input those features into a categorization method [65]. Khan et al. [66] provided a strategy for identifying and categorizing melanoma and nevi. The author started by attempting to eliminate the noise using a Gaussian filter. For lesion segmentation, K-mean clustering was used. A hybrid super feature vector was used to extract textural and color data. Then, support vector machines, often known as SVMs, were used for the categorization procedure. The accuracy of the suggested approach was 96% when applied to the ERMIS dataset. Deep learning (DL) and handmade features were brought together in the novel approach introduced by Filali and colleagues [67]. The created technique attained an accuracy of 98% on the Ph2 dataset; however, it only achieved an accuracy of 87.8% on the ISIC challenge dataset. A technique that was based on measuring the similarity of features was utilized by Hu et al. [68], and SVM was then used to perform the categorization. Abbas et al. [69] introduced a five-layer technique called “DermoDeep” to discern between benign moles, known as nevi, and malignant moles, known as melanoma. Their approach combined visual characteristics with a five-layer model to get the most accurate classifications possible. Dalila et al. [70] retrieved three different kinds of data, including texture, geometrical qualities, and color, and then used ant colony-based segmentation to choose the most useful features. After that, an ANN was used for the categorization process. A strategy provided by Almansour et al. in [71] included the extraction of textual characteristics, followed by the use of an SVM as a classifier. On 227 different photos, the given approach accomplished an accuracy of 90%. Pham et al. [72] extracted ROIs using image enhancement methods to get their results. After that, the SVM was used to classify the photos that had been preprocessed. The accuracy that was achieved was 87.2%. Yu et al. [73] presented a strategy to improve the pictures to extract ROIs and used a deep residual method to categorize the imageries. The accuracy of the suggested scheme was determined to be 85.5% after testing. Recent efforts in the cancer research domain have engrossed the categorization of melanomas using deep DL, archived with an accuracy of 86.54%. A deep CNN design was suggested by Rokhana et al. [74] to classify melanoma dermoscopy pictures into benign and malignant skin lesions. On the ISIC-archive repository, the provided method was reviewed for its effectiveness. The strategy that was suggested achieved a sensitivity of 91.97%, an accuracy of 84.76%, and a specificity of 78.71%. The classification strategy employed by Xie et al. [75] was based on the ensemble model. An ensemble model that is built on three different classifiers was created by Liberman et al. [76] to identify mole pictures as either non-melanomas or melanomas. Zhou and colleagues [77] introduced a novel approach that is founded on spiking NNs with time-dependent spike plasticity. A DCNN architecture was constructed by Hosny et al. [78] for melanoma categorization. The methodology that will be given was evaluated using three distinct datasets. The technique employed by Mukherjee et al. [79] was called CNN malignant lesions detection (CMLD), and it was based on CNN. In the MED-NODE and Dermofit datasets, the created model attained an accuracy of 90.14% and 90.58%, respectively. Deep neural networks were used in the method that Esteva et al. [80] developed to identify skin problems at an early stage and categorize skin cancer. Cakmak et al. [81] provided a model for detecting melanoma that was built on a deep NN and given the name Nasnet Mobile. The provided method was tested for its effectiveness on the HAM10000 dataset. To address the issue of unequal class composition, many augmentation strategies were used. The recommended approach’s accuracy was 89.20% without DA and 97.90% with DA when used with the Nasnet-Mobile network. Brinker et al. [20] categorized the SC as either melanoma or nevi by employing a pre-trained architecture that was given the label ResNet50. The suggested model achieved a sensitivity ratio of 77.9% and a specificity ratio of 82.3%, correspondingly. Han et al. [82] used the ResNet152 approach to categorize several skin lesions. Melanoma, seborrheic keratosis, and nevi had a specificity of 87.63% and a mean sensitivity of 88.2%, respectively, when it came to being diagnosed with the condition. When attempting to categorize skin lesions, Hosny et al. [83] modified AlexNet such that its last three layers consisted of fully linked layers, softmax, and an output layer. The accuracy accomplished by the recommended technique was 96.86 percent. When classifying skin lesions, Esteva et al. [84] made use of a pre-trained model that was given the name Inception-v3. By using several augmentation methods, they expanded the testing dataset.

## 3. Materials and Methods

### 3.1. Dataset Description

We used an SC dataset that was accessible to the general audience. The dataset was being analyzed for the following stated motives: (1) to increase the dataset scope for training drives; (2) to reduce overfitting and unfairness; and (3) to contain two classifications (Cancer, and Non-cancer). Integrating the datasets was another factor that contributed to the model’s efficacy. The dataset contains 288 photos, of which 84 are cancerous and 204 are not cancerous (https://www.kaggle.com/datasets/kylegraupe/skin-cancer-binary-classification-dataset; retrieved on 16 June 2023). The photos are saved in JPG format and vary in the number of pixels they possess.

### 3.2. Methodology

#### 3.2.1. Motivation

It is possible that DL algorithms may be used successfully to identify skin cancer. DL methods have been used for similar problems, such as breast cancer classification [85], Parkinson’s ailment categorization, and the detection of pneumonia utilizing chest radiograph images. Inspired by the success of the DL-built network in identifying BC in specific photos, this research offers a SkinNetX model for identifying and categorizing skin cancer. The study’s ultimate goal is to recommend a DL model to better identify skin cancer from dermatological pictures. The proposed model uses the following data to determine the depth and input picture resolution: A deeper DL-based model is believed to boost the model’s categorization effectiveness by encapsulating further nuanced and relevant deep information. To further improve accuracy, several DL-based models have used depth scaling. While expanding the network’s depth might potentially enhance accuracy, doing so comes at the rate of an upsurge in computing intricacy. DL-based models use high-resolution input images because of the improved performance they provide. Images with resolutions between 224 by 224 to 299 by 299 may be detected by DL models, while models with a higher resolution often have superior performance. Similarly, the proposed model includes 24 layers and can handle pictures with a resolution of 224 × 224. Based on the limitations of the available computing resources, we choose the SkinNetX architecture and the appropriate input picture size.

#### 3.2.2. Image Augmentation and Preprocessing

Normalizing images is a crucial step before feeding them into a CNN model. The photos in both datasets have been scaled to accommodate the varying input sizes of the various models. Algorithms based on DL are notorious for their high data needs. An immense volume of data is necessary to train DL algorithms to a high level of accuracy. Because of their dependence on data, they are challenging to use in under-resourced areas or fields. Not all domains have enough data to train a DL system, but we were able to fill in the blanks using the DA procedure developed in this paper. All of the images in the training set were randomly rotated by an angle 30 degrees and translated up to 30 pixels in both the vertical and horizontal directions. Random translations between [0.9 and 1.1] were used to produce more pictures. Consequently, a DL algorithm might be trained on very little data to get satisfactory outcomes without overfitting the data’s widely held class.

#### 3.2.3. Deep Learning (DL)

Machine learning methods are comparable to AI strategies since they attempt to mimic human learning processes. Conventional machine learning algorithms require preprocessing activities such as feature extraction and a rigorous feature selection procedure before beginning the learning and classification stage. Whether or not these methods are successful depends heavily on the qualities used to make distinctions across classes. Unlike traditional ML approaches, DL provides for the automated learning of feature sets for different responsibilities [20]. The field of data science would be incomplete without DL, which is also a component of predicting and statistics. To interpret visual data, a DNN known as a CNN, is employed. A CNN uses a DL approach called weighted convolutional recurrent networks to discriminate between objects in an input picture. Due to its remarkable accuracy, CNNs can classify and identify images [86,87].

#### 3.2.4. SkinNetX Model

**Figure 2:**
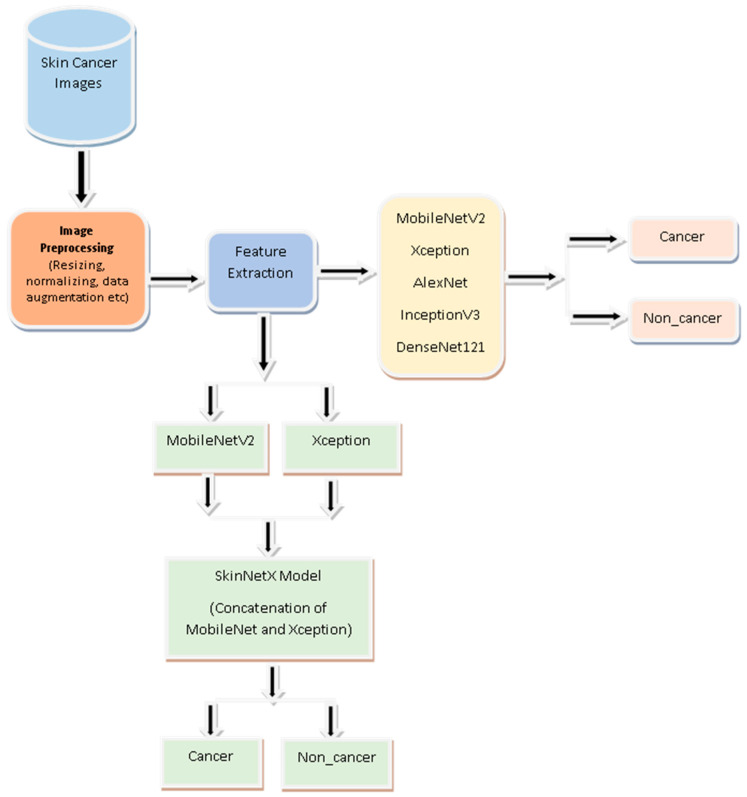
Proposed Skin cancer detection System.

Image classification is the emphasis of the proposed approach, which is a deep learning approach. Figure 2 demonstrates the abstract view of the suggested SkinNetX approach comprising three core components. The recommended technique is more complicated than a conventional CNN.

Fusing MobileNetV2 with Xception creates a more robust and precise model than any of its components could achieve on their own. The “ImageNet” dataset provides the MobileNetV2 model with its first pre-trained weights, allowing it to use the information gained through analyzing a considerable body of photos. Layers are added to the model after the output has been generated, including a global average pooling layer, a dense layer with ReLU activation, and a dropout layer. However, MobileNetV2’s final layers are left out.

The Xception model is likewise set up using “ImageNet” pre-trained weights, and the last fully linked layer is left out. Like MobileNetV2 output, it passes through many additional processing stages. Combining the two models’ results may teach us more generalizable characteristics about the pictures we feed them. After the combined output is sent through the concatenated layer, a dense layer with softmax activation is added for classification.

The performance of both the MobileNetV2 and Xception models may be improved by making some layers non-trainable (frozen) and hence unmodifiable throughout the training process. This strategy helps customize the model to meet the needs of a given picture classification challenge while using the previously acquired information.

An Adam optimizer, a loss function based on categorical cross-entropy, and an accuracy measure are used to create the approach. Then, the process is trained on a labeled dataset to achieve the highest possible accuracy while minimizing the loss. For context, a summary of the model is printed out, detailing its structure in terms of layers and the number of parameters.

Finally, an early stopping callback is implemented to monitor the validation loss and bring back the optimal weights if the loss does not decrease after a certain number of iterations. Keeping the top-performing model like this helps avoid overfitting. The attributes of the SkinNetX is shown in Table 1.

**Figure 3:**
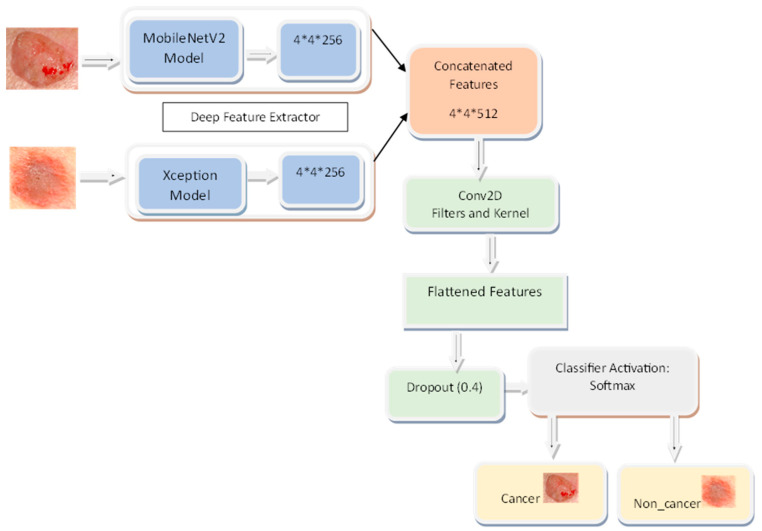
SkinNetX Architecture.

The suggested model has many layers that cooperate in the picture categorization task. The MobileNetV2 convolutional layer takes input photos and generates their features. After this layer, we apply GlobalAveragePooling2D to the feature maps to make them more manageable in terms of space. The 256-filter dense layers are fully connected layers that can pick up sophisticated data patterns. Overfitting may be avoided using the dropout layer, which sets a percentage of input units to 0 at random throughout the training procedure. The results from the MobileNetV2 and Xception levels are combined in the concatenate layer. The Xception layer is a convolutional layer that, like MobileNetV2, helps to extract more features from the photos. The last dense layer, equipped with 512 filters, acquires additional pattern knowledge from the combined data. A dense layer with two filters generates the output probabilities for image classification and utilizes the softmax activation function. Together, MobileNetV2 and Xception contribute to the overall accuracy of this model’s picture classification. Figure 3 shows the architecture of the proposed SkinNetX model.

### 3.3. Transfer Learning (TL)

DCNN approaches are still widely employed in contemporary research, providing novel solutions for skin cancer detection. However, a widespread problem is a deficiency in the volume of training data necessary to operate deep CNN models successfully [88]. A procedure identified as transfer learning (TL) is used as a solution because compiling a comprehensive dataset on skin cancer is time-consuming [88]. The CNN models used in TL are first trained on big datasets, and then they are fine-tuned using smaller datasets that the user specifies. The minute training data are absent, and this method is advantageous because of its effectiveness. Similar to training from the beginning, the amount of time spent on training is drastically reduced when using pre-trained models that already understand fundamental characteristics. Memory and computational resources are also reduced to a minimum by TL. Pre-trained algorithms, such as those trained on the ImageNet dataset, which includes millions of photos spanning various categories, are used extensively in different TL techniques.

On the other hand, the pre-trained technique used in this investigation was trained not on the ImageNet dataset but on skin cancer ultrasound pictures. TL is applied extensively in a variation of computer vision responsibilities, including the diagnosis of skin cancer. During the study, nine distinct CNN models were calibrated using the information about skin cancer. For SC recognition and classification, five pre-trained CNN models were used. Table 2 contains detailed information on the number of parameters and layers included in the various CNN designs.

#### 3.3.1. Xception Model

The Xception network has taken up the duties formerly performed by Inception. XceptionNet is the name given to the extreme form of Inception [89]. In the XceptionNet network, conventional convolution layers have been swapped out for depthwise separable convolution layers. CNN feature maps enable decoupling spatial and cross-channel correlations, while XceptionNet’s mapping of spatial and cross-channel correlations is included in the network’s core functionality. XceptionNet eventually superseded the main architecture of Inception [90]. The XceptionNet model’s total of 36 convolution layers is capable of being segmented into a total of 14 separate modules. After the initial and final layers have been eliminated, there is still a continuous relationship between each of the layers that remain. The original image must first be translated into determining the probability contained across several input channels to provide a unified image [90]. The subsequent strategy takes advantage of the 11 depthwise convolutions. As an alternative to three-dimensional maps, displays showing relationships could be used.

#### 3.3.2. MobileNetV2

The MobileNetV2 model is a competent and lightweight CNN technique developed specifically for mobile and embedded settings [91]. By separating the spatial convolution from the depthwise convolution, this network may reduce computational costs without sacrificing performance. The MobileNetV2 model comprises 88 discrete levels. Both convolutional and fully linked layers are present [65]. About 3.5 million parameters make up the MobileNetV2 model. The weights and biases acquired during training are reflected in these parameters. The MobileNetV2 model occupies approximately 14 MB of space. When deploying the model on resource-constrained devices, it is vital to consider the size shown by this metric, as it shows the memory needed to hold the model’s parameters [92]. In summary, the MobileNetV2 model strikes a nice compromise between model size and performance, making it appropriate for applications with limited computing resources, such as mobile devices or embedded systems.

#### 3.3.3. AlexNet Model

AlexNet claims that there are 11 tiers to the entire system. The substantial amount of underlying network layers facilitates feature extraction. Furthermore, a wide range of elements operates to improve effectiveness generally. AlexNet’s initial stage is a convolutional one. The final layer is a convolution layer following the maximum pooling and regularizing layers. When the softmax layer is used, categorization is complete [93].

#### 3.3.4. DenseNet121 Model

DenseNet121 was first released in 2017 by Huang et al. [87] Layer connections in DenseNet models tend to be many. DenseNet121 gets its name from having 121 layers. DenseNet fixes the problem of the diminishing gradient and boosts the network-wide gradient flow. In conventional CNN architectures, data can only be fed in from the layer above it. All inputs from previous DenseNet layers are available to the current layer. This structure permits dense or skipped connections between layers, facilitating direct information flow and gradient propagation. As a result, DenseNet can make more use of layer data and boost feature reuse.

The convolutional layers of DenseNet121’s dense blocks are many. Each deep layer’s feature maps are merged into one massive map that is then passed on to the next. The feature maps and spatial dimensions suffer when transition layers are present between dense blocks. These transform layers use pooling and 1 × 1 convolutional layers to compact feature maps. DenseNet121 uses a softmax activation layer, a fully connected layer, and a global average pooling layer as its final layers. The fully connected layer provides classification after the global average pooling layer reduces the spatial dimensions to a vector. DenseNet topologies are widely used because they are more efficient and have less overfitting and gradient flow issues. DenseNet121 has shown promising results in image classification, object recognition, and segmentation benchmark datasets [94].

#### 3.3.5. InceptionV3 Model

In 2015, scientists at Google developed InceptionV3. Images may be sorted into categories and recognized using inception models. ImageNet Large-Scale Visual Recognition Challenge (ILSVRC) results showed that InceptionV3 was the best system available at its release. The Inception algorithm compensates for the trade-off between computing speed and network depth. An Inception module is used in InceptionV3 to mix convolutional filters of varying sizes. These filters collect data at various scales, allowing the network to better use available processing power [95].

As the network becomes more profound, the stacked Inception modules in InceptionV3 can pick up on more abstract features. Gradient flow is improved during training using convolutional layers, pooling layers, fully connected layers, and auxiliary classifiers. Images may be sorted, identified, and segmented using InceptionV3. Its effectiveness and efficiency help study and implement deep learning [95].

### 3.4. Hyperparameter Setting

Because hyperparameters influence the learning process and are the fundamental component of the model, they must be provided before any model can be trained. This is because hyperparameters are the model’s most significant component. Finding the proper criteria to meet may be accomplished in several different ways. We use ratios of 80/10/10 to choose which skin cancer pictures will be used for training and validation and which will be used for testing. The batch size is the number of training samples tallied in a single forward and backward pass. When the batch size increases, the amount of memory space needed also increases.

A hyperparameter identified as the learning rate (LR) determines the degree to which the weights of the suggested approaches are modified in response to changes in the loss gradient. We make our way down the hill more leisurely as the value decreases. A low LR might be an excellent option to guarantee that we do not pass over specific regions. However, it might accelerate convergence in the long run, especially if we get stuck on a plateau area. This is especially true if we cannot move off the plateau. The number of times the machine learning algorithm has iteratively gone through the whole training dataset is referred to as the “epoch” in this context. Batches are a standard method for organizing datasets, mainly when the total quantity of data to be processed is substantial. Some people use the word “iteration” to refer to one batch processed by the model; however, this is an imprecise term. When training a neural network with sample data, one of the most significant challenges is avoiding overfitting. When preparing a NN approach with more epochs than required, the training process will mostly learn patterns specific to the trial data used for training. Because of this, the model cannot work correctly when applied to a new dataset.

The performance of this technique is satisfactory on the training set (the data from the samples) but unsatisfactory on the test set. In other words, if the model is overadjusted to the data used for training, it will lose its capacity to generalize. Mini-batch regression is a variant of the technique identified as gradient descent in which the training dataset is divided into fewer batches. These batches are then utilized to calculate the technique error and update the coefficients. Mini-batch gradient descent is an example of an iteration of the gradient descent process. By adding up the gradient across each mini-batch, implementations can further limit the gradient’s variation.

To strike a balance between the efficiency of batch gradient descent (BGD) and the robustness of stochastic gradient descent, the miniature BGD technique was developed. In DL, gradient descent is the preferred method since it yields several benefits.

We employed a grid search technique to determine the optimum hyperparameters for the recommended DL model, which gives a high level of accuracy with a minimal margin for error. Stochastic gradient descent, often known as SGD, was used throughout the training process of DL models that had previously been trained with TL. We worked with a mini-batch size of 32 images and a learning rate of 0.001 per picture. In addition, to prevent overfitting, each DL model was trained for one hundred epochs before it was put through its pace in the TL tests designed to recognize and categorize various types of SC. All our trials were conducted on a computer that had an Intel (R) Core (TM) i5 and 16 gigabytes of random-access memory (RAM). Jupiter Notebook in Anaconda was the software that we used for the implementation. The optimal values for each of the categorization experiment’s parameters are shown in Table 3.

The hyperparameters outlined in Table 3 are essential to the training and to the overall performance of the SkinNetX model suggested in this study. The use of a learning rate of 0.0001 signifies the adoption of a fine-tuning methodology, which mitigates substantial modifications to the model’s parameters and the risk of overfitting when using pre-trained networks. Stochastic gradient descent (SGD) as the optimizer confers advantages to the model due to its simplicity and effective handling of big datasets. Training the model over 100 epochs makes it possible to run through the whole dataset many times, facilitating the acquisition of knowledge from a wide range of samples and perhaps achieving convergence toward an optimal answer. The rectified linear unit (ReLU) activation function is widely favored because of its straightforwardness and computational effectiveness. By incorporating nonlinearity into the hidden layers of the model, ReLU enables neural networks to acquire intricate patterns successfully. Furthermore, implementing early stopping with a patience value of 80 guarantees that the training procedure will be terminated if there is a lack of substantial performance improvement over a prolonged duration. This approach serves to mitigate the risk of overfitting and conserves computing resources.

The hyperparameters generally exhibit a well-calibrated configuration to achieve a harmonious equilibrium between efficient learning from the available data and mitigating the risk of overfitting. The selection of hyperparameters demonstrates a meticulous evaluation of the model’s structure and the dataset’s attributes. However, it is essential to recognize that hyperparameter tuning is often iterative and exploratory. The efficacy of these selections is typically confirmed by testing conducted on a particular dataset. Additional information about the hyperparameter-tuning methods, including cross-validation or other optimization strategies, would provide a deeper understanding of the resilience of the proposed SkinNetX model’s proposed configuration.

### 3.5. Performance Evaluation Metrics

Accuracy, precision, recall, and the F1-score were the metrics the study relied on to assess the effectiveness of the models applied in this investigation. The following formula can determine these:

(1) $$Accuracy=\frac{TP+TN}{TP+FP+TN+FN}$$

(2) $$Precision=\frac{TP}{TP+FP}$$

(3) $$Recall=\frac{FN}{FN+TP}$$

(4) $$F1-score=2*\;\frac{\left(precision\;\times \;recall\right)}{\left(precision+recall\right)}$$

The term “true positive” (TP) refers to the positive data that have been adequately anticipated and estimated. The most significant value is found along the diagonal.

True negative (TN): The analysis of the negative data shows that the data are, in fact, negative. A TN is the total of all the values in the confusion matrix, except for the row and column that correspond to the related class.

A false positive (FP) occurs when data that should have been negative are evaluated as positive. It is the sum of all the values in the column that pertains to each class, except for TP.

The interpretation of positive facts as having a negative impact is one example of a false negative. It is the sum of all the values in the row that pertain to each class, except for TP.

## 4. Results and Discussion

### 4.1. Performance Evaluation of SkinNetX Model

The performance of the suggested DL model in terms of detection (two-class classification) is being evaluated with the help of photos of skin cancer in this experiment. The dataset was partitioned into training, validation, and testing sets for this experiment. For this experiment, 15% of the data was used for testing the model, 10% for validating the model, and 75% for training. To be more exact, we utilized all 408 SC dermatoscopy photos, of which 311 were used for training, 56 for validation, and 41 for testing. There were 204 cancer images and 204 non-cancer (benign) images. The parameters supplied in Table 1 are used in the training set to practice the SC detection and classification provided by the suggested framework. Throughout the 12 epochs, the proposed DL model went through a total of 1200 iterations, with an average of 100 iterations occurring throughout each epoch.

**Figure 4:**
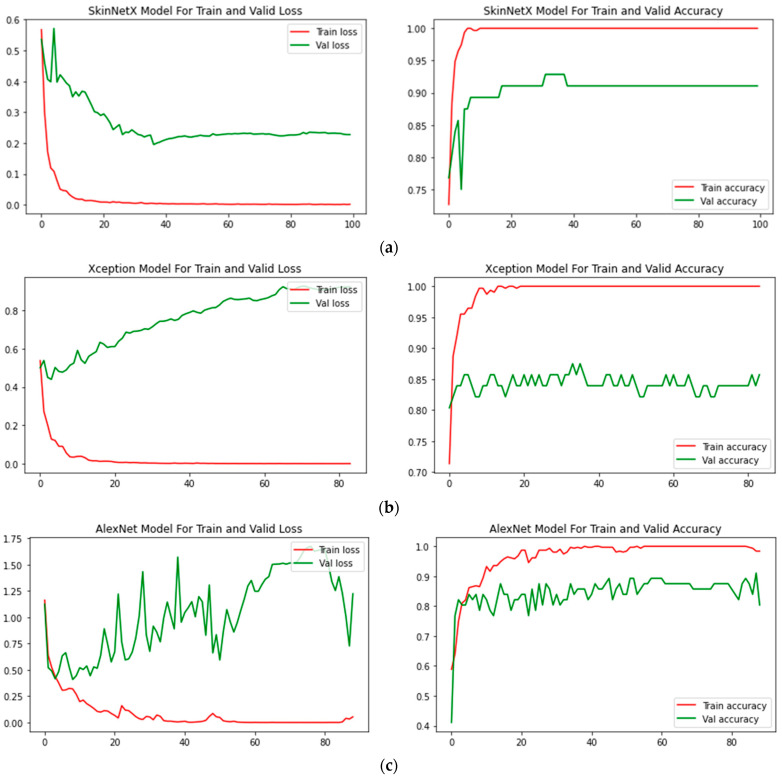

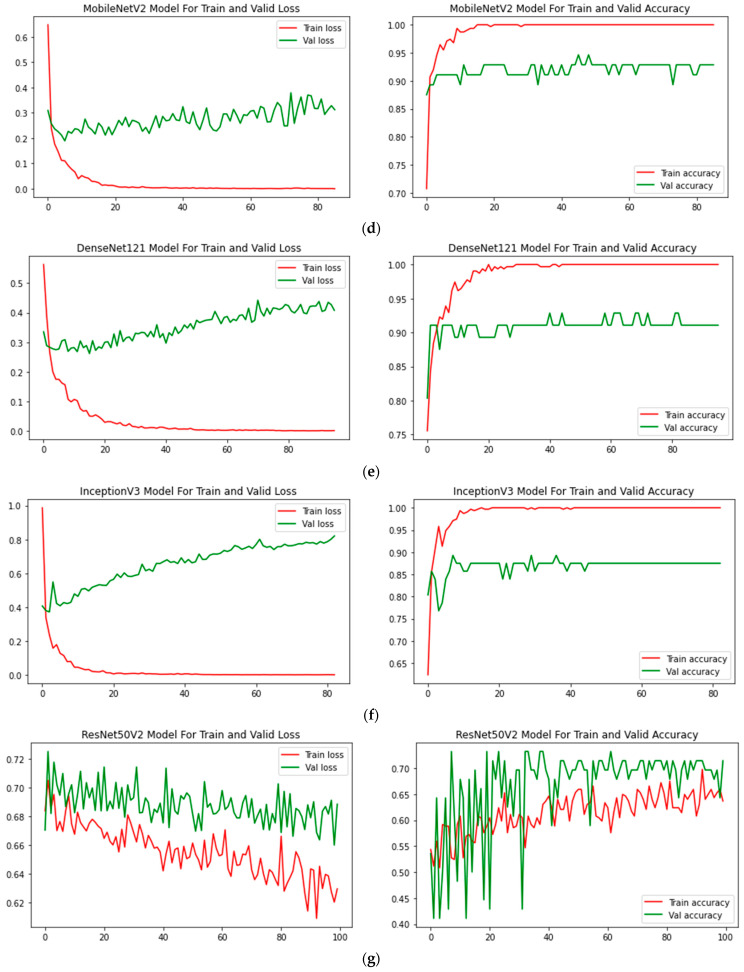
(**a**) SkinNetX; (**b**) Xception; (**c**) AlexNet; (**d**) MobileNetV2; (**e**) DenseNet121; (**f**) InceptionV3; (**g**) ResNet50V2 Loss and accuracy graphs.

**Figure 5:**
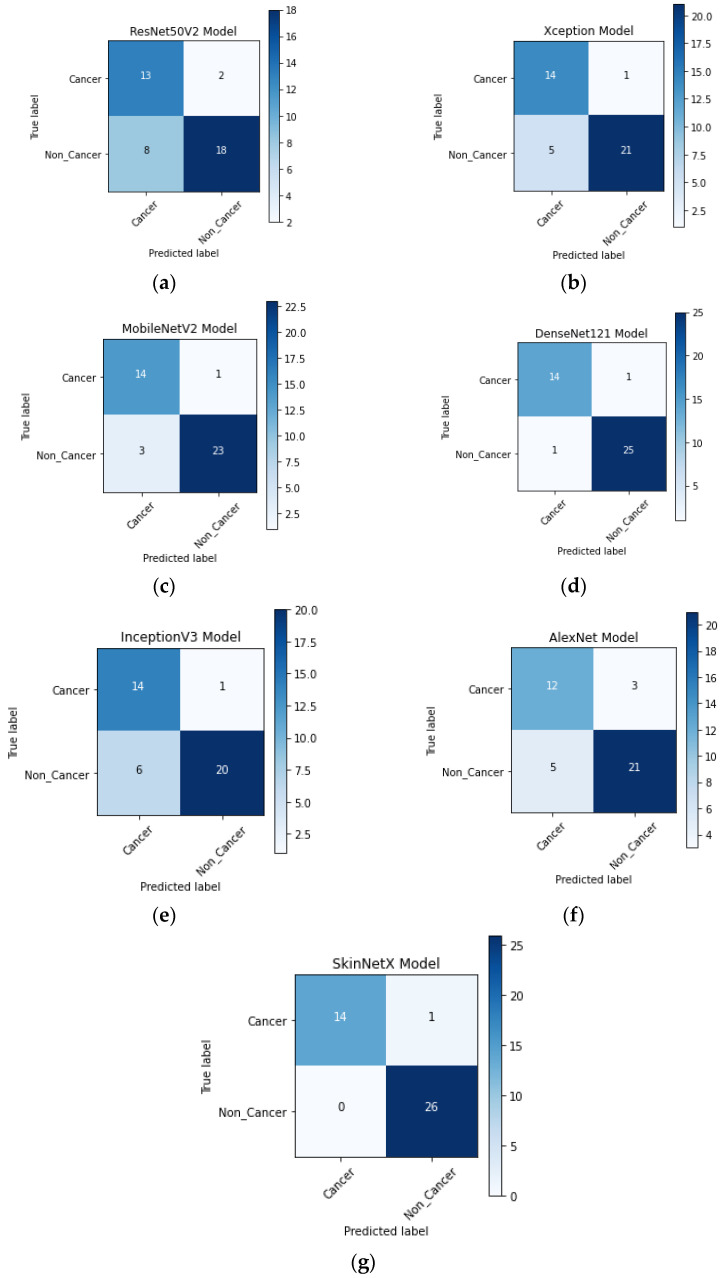
(**a**) ResNet50V2; (**b**) Xception; (**c**) AlexNet; (**d**) MobileNetV2; (**e**) DenseNet121; (**f**) InceptionV3; (**g**) SkinNetX confusion matrix on the test dataset.

**Figure 6:**
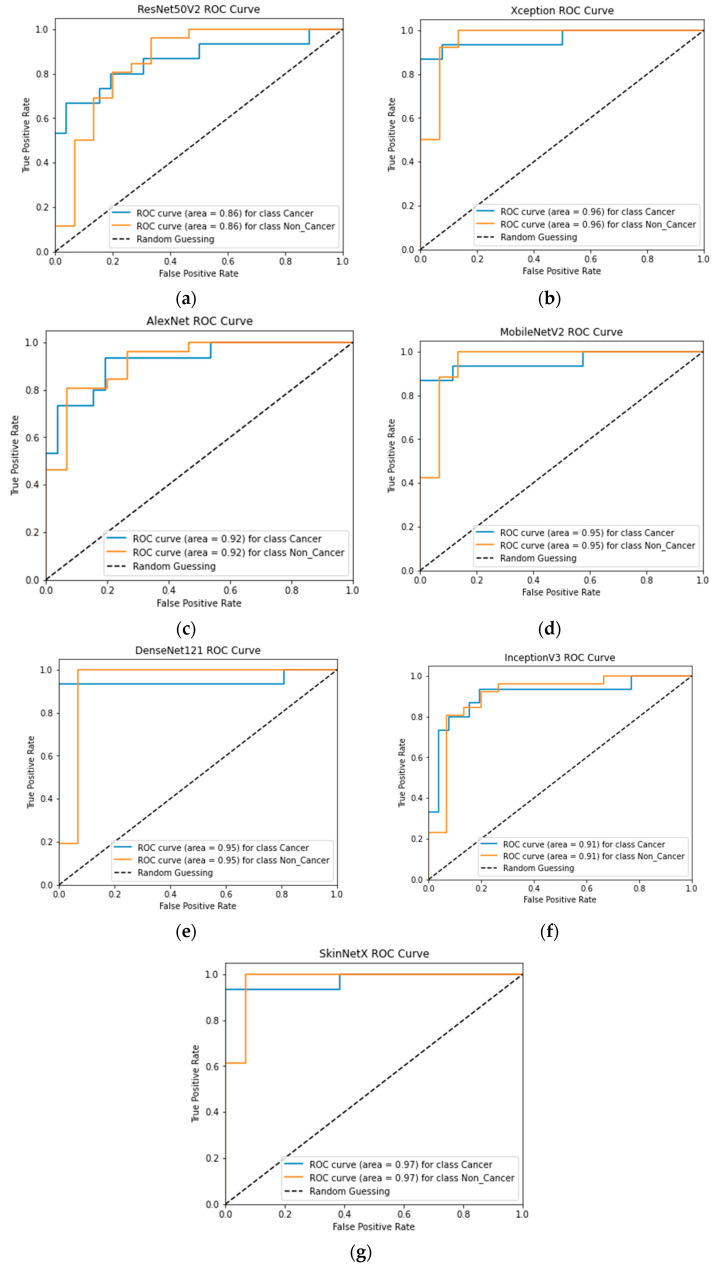
(**a**) ResNet50V2; (**b**) Xception; (**c**) MobileNetV2; (**d**) AlexNet; (**e**) DenseNet121; (**f**) InceptionV3; (**g**) SkinNetX ROC-AUC Graph Curves.

**Figure 7:**
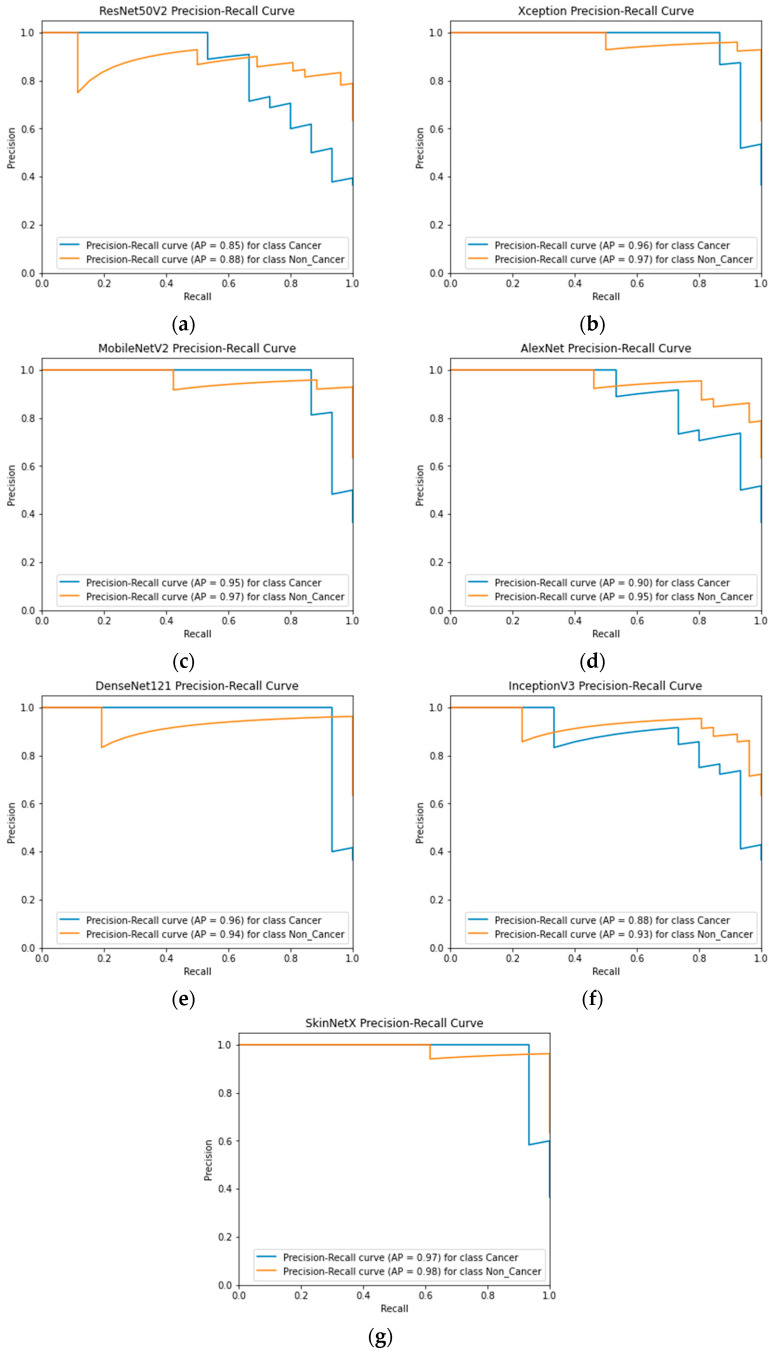
(**a**) ResNet50V2; (**b**) Xception; (**c**) MobileNetV2; (**d**) AlexNet; (**e**) DenseNet121; (**f**) InceptionV3; (**g**) SkinNetX Precision–Recall Graph Curves.

**Figure 8:**
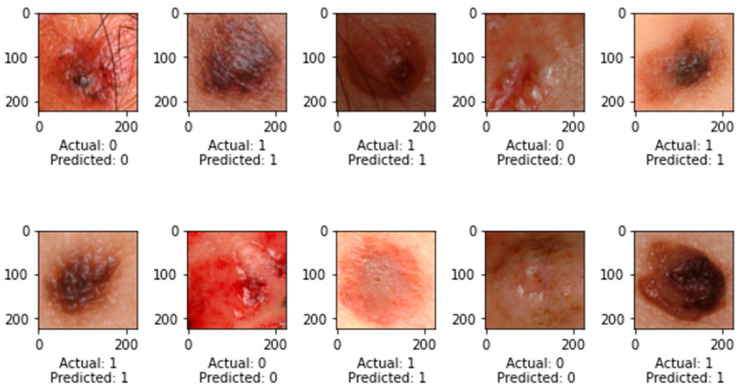
Prediction on Test Dataset.

At epoch 100, the recommended SkinNetX obtained maximum classification accuracy of 97.56%, a precision of 93.33%, a recall of 100%, and F1-score values of 96.55%, proving the efficacy of our approach in identifying SC. To further demonstrate the effectiveness of the suggested strategy during training and validation, we have plotted accuracy and loss in Figure 3. The loss function reflects the accuracy with which the framework makes predictions. Starting at epoch 12, our model’s loss and accuracy remain approximately the same for the training process, and starting at epoch 39, our model has stayed roughly the same for the validation process. In other words, it is still superior at predicting SC at epochs lower than 100. Figure 4 shows the suggested DL methods and the training and validation graphs of the already-trained models. Figure 5 displays the SkinNetX and state-of-the-art pre-trained DL detections’ confusion matrices from the testing phase. The recommended approach was classified incorrectly with just two images. Figure 6 shows the ROC-AUC curve for all the implemented techniques, and it can be seen that the proposed SkinNetX technique outperformed with an AUC of 0.97 for both cancer and non-cancer classes. The second best is Xception with an AUC of 0.95 for both data classes. MobileNetV2 and DenseNet121 both had the third-best AUC value of 0.95. The precision–recall curve for all the implemented techniques is shown in Figure 7 with SkinNetX having the highest value of averaged precision (AP), thereby outperforming the other pre-trained techniques. Figure 8 shows the prediction of the test dataset, and it is seen that out of the ten (10) samples predicted, all of them were predicted correctly.

Our proposed technique may improve SC recognition and categorization from dermatoscopy pictures. Our suggested method successfully represents the dermatoscopy picture by extracting the most distinctive, resilient, and sophisticated deep characteristics for precise and reliable detection. The recommended approach is straightforward because of its fully integrated learning architecture, eliminating the need for a separate feature extraction step.

### 4.2. Comparative Evaluation Using Cutting-Edge Pre-Trained DL Models

Overfitting issues are frequently encountered in DL methods during evaluation with limited training and testing images; employing TL techniques and fine-tuning may assist in mitigating this issue. This often occurred because it was more effective for assessing with fewer but diverse instances of training and testing pictures. All TL simulations were trained and validated utilizing the equivalent TL parameters as listed in Table 4 for SC identification and classification. These parameters were used throughout the training and validation processes. We used 408 dermatoscopy photos of BC to identify BC correctly. As can be seen in Table 4, which provides a summary of the individual findings obtained by several TL algorithms while categorizing SC pictures, every TL classifier was successful in producing acceptable results. We investigated and evaluated the TL algorithms by using evaluation metrics such as accuracy, precision, recall, an area under the curve (AUC), and F1-measure.

For this study, we split the dataset into three parts—training, validation, and testing—using 75% of the data to develop models, 10% for validation, and 15% for testing. The suggested study comprised a variety of deep learning-based categorization methods, including Alexnet, MobileNetV2, InceptionV3, Xception, DenseNet121, and the proposed DL. As can be seen in Figure 4 of the recommended approach’s confusion matrix, the score is high across the board for SC categories.

In terms of accuracy, it is seen that SkinNetX performed at a 97.56% level, ResNet50V2 performed at a 77.50% level, and AlexNet performed at an 80.49% level, making it the second-worst performer. Regarding accuracy, DenseNet121 came in second with a score of 95.12%. The accuracy of MobileNetV2 was 90.24%, Xception was 85.37, and InceptionV3 was 82.49%. The results are also superior to those obtained by using already-trained approaches. Table 4 shows the model classification results in the same period. Regarding accuracy, precision, recall, F1-measure, and the area under the curve (AUC), the SkinNetX model is superior to all other pre-trained models. The SkinNetX has the least misclassification rate of 0.0244, followed by DenseNet121 with the second-least misclassification of 0.0488 and MobileNetV2 with the third-least misclassification of 0.0976. The accuracy and loss values are shown in Figure 4 when the proposed model is trained and verified. The graph in Figure 4 illustrates that, as indicated in Table 4, the custom model provides exceptionally high accuracy results.

### 4.3. Comparative Studies with Recent Research

In this study, we compared the performance of the best deep neural network, SkinNetX, with other methods for classifying SC tumors into cancerous and non-cancerous categories. We compared the work that was suggested to state-of-the-art deep learning (DL) approaches [96,97,98] in more detail.

Tembhurne et al. [96] developed an innovative method for extracting characteristics from photographs of the skin, which was then used in skin cancer diagnosis. They combined the Cnoyrlet transform with LBP histograms and VGG19 in their analysis. They were able to achieve an accuracy level of 93%. A unique technique for the categorization of skin lesions in metadata-embedded photos using a deep CNN (Inception-ResNet-v2) was presented by Mehr and Ameri [97] in 2022. The suggested method successfully distinguished between malignant and benign lesions with a rate of accuracy that reached 94.5%. A lightweight skin cancer classification model based on deep learning approaches was developed by Huang et al. [98] in 2021 to assist first-line medical treatment. When we used the DenseNet 121 model for classification, we achieved an accuracy of 89.5% for the binary categories (benign vs. malignant).

This part offers a comprehensive analysis and comparison of numerous tactics based on their accuracy. Table 5 includes accuracy on its list of performance parameters, although it is the research parameter employed in every relevant study the most. As far as we know, the recommended DL approach surpasses the previously published state-of-the-art methods. The capacity of the suggested approach to extract more stable and distinguishable deep features for classification is one factor contributing to achieving the best results. In addition to that, we used a dataset that was combined and balanced. The comparison outcomes revealed that the presented strategy was more successful than the other strategies. In addition, the classification process, which requires more intricate computing, necessitated hand-crafted engineering. Regarding accuracy, the recommended model performed better than the presented strategies, as shown in Table 5.

## 5. Discussion

The result was reached by analyzing the validation data, which included two skin cancer datasets. To put the deep models that were used in this study into practice, we relied on the Keras library [99]. Due to its flexibility to operate on top of other deep learning frameworks, such as TensorFlow, Keras is becoming more popular. Models are trained using a computer system with 16 gigabytes of random-access memory (RAM), a Corei7 processor, and a one-terabyte hard drive.

We examined the performance of seven different models, including ResNet50V2, Xception, AlexNet, MobileNetV2, DenseNet121, InceptionV3, and SkinNetX, in categorizing skin cancer into cancer and non-cancer categories. These models are listed in alphabetical order below. It was discovered that ResNet50V2, Xception, AlexNet, MobileNetV2, DenseNet121, InceptionV3, and SkinNetX each had a categorical accuracy of 77.50%, 85.37%, 80.49%, 90.24%, 95.12%, and 82.93%, respectively. SkinNetX had a categorical accuracy of 97.56%. The SkinNetX model has been shown to have the highest accuracy.

Figure 7 displays, for each of the seven models, the training–validation accuracy and the training–validation loss curves. At the beginning of the training process, during the first few epochs, the validation accuracy is higher than the training accuracy, or the training loss is lower than the validation loss. There are a few different ways that this might be explained. In the first place, since we included the dropout layer in the architecture while fine-tuning the model to make our system less susceptible to over-fitting, these dropout layers block the neurons when the model is being trained to simplify the model. When testing a model in Keras, dropout layers are turned off throughout the process. This gives the network access to all of the computing capacity it needs to make a prediction, which may result in improved training accuracy for a few epochs. At the same time, the model is being evaluated [100]. Second, the training loss is calculated by taking the average of the losses occurring over all training data batches. The loss over the most recent batches is often larger when compared with the loss over the batches that began an epoch since the model is developing over time. In a different approach, the training loss for a model is determined after each epoch, which often results in a smaller loss. This can potentially lead to a smaller training loss than the validation loss.

A weighted average of recall, precision, and F1-score is also examined to verify the performance of models with the number of pictures for each class of validation data. This evaluation is conducted to check how well the models do their job. We performed some calculations, and the results showed that SkinNetX had a weighted average recall, precision, and F1-score of 100%, 93.33%, and 96.55%, accordingly. Similarly, the weighted averages of recall, accuracy, and F1-score for the models ResNet50V2, Xception, AlexNet, MobileNetV2, and DenseNet121, as well as InceptionV3, were analyzed. The outcomes of the seven distinct models that were used for this study are presented in Table 4. These results include accuracy, a weighted average of recall, precision, and F1-score.

The SkinNetX model, as suggested, demonstrated improved predictive accuracy within the medical field. This improvement may be attributed to its capacity to extract more effective feature representations from pictures of skin lesions. This capability is aided by using transfer learning and fine-tuning techniques applied to preexisting models. The enhanced applicability of the model to various kinds of skin lesions may be attributed to its capacity to withstand variations in medical imaging datasets, such as changes in lighting conditions and picture quality. Using an expanded and heterogeneous dataset, in conjunction with meticulous hyperparameter optimization, might have enhanced its overall performance.

The suggested SkinNetX has notable benefits, namely in its ability to attain a commendable accuracy level of 97.56% when categorizing tumors associated with skin cancer. Ensuring trustworthy and precise diagnoses is paramount in medical applications, necessitating high precision. SkinNetX demonstrated superior performance compared with other pre-trained models, such as ResNet50V2, AlexNet, DenseNet121, MobileNetV2, Xception, and InceptionV3, as well as previously reported cutting-edge methodologies. This suggests that SkinNetX has promise for yielding superior outcomes compared with current methods. Transfer learning (TL) enables SkinNetX to exploit acquired knowledge from diverse tasks and datasets, hence aiding in alleviating overfitting concerns and diminishing the need for extensive training datasets. The model is said to extract consistent and discernible deep characteristics for categorization. This has the potential to enhance the differentiation between malignant and benign skin lesions, enhancing the overall efficacy of the model. Finally, a balanced dataset may contribute to the model being trained on representative samples from many classes, improving its capacity to generalize to novel and unfamiliar data.

One disadvantage of the suggested approach is that it relies on pre-trained deep learning algorithms. As a result, implementing the system requires substantial computing resources for training and inference. This constraint hinders the adoption of the approach in resource-constrained environments and low-end devices. There exists apprehension over the capacity to extrapolate findings to novel and unfamiliar skin lesions that lie outside the confines of the available datasets.

## 6. Conclusions

A unique SkinNetX deep learning model for SC detection and classification is presented in this study. A total of 157 layers are included in the architecture that has been delivered, some of which include convolutional layers, dense layers, and a fully linked layer. According to our observations, the suggested model achieved the best classification accuracy of 97.56%. In addition, we examined how well a few other deep learning models worked, and the experiment results revealed that our model performed far better than the others. In addition to that, we used the test dataset to verify the model that was presented. The suggested model achieved the most significant possible level of accuracy throughout testing, which was 97.56%. The TL method used in this research for SC detection employed four pre-trained DL models.

On a standard dataset that included 204 cancer and 204 non-cancer (benign) dermatoscopy pictures, we tested the efficacy of five different DL-based models and compared their results. A disadvantage of this study is that there are only a limited number of photos in the SC dermatoscopy imaging dataset that is publicly accessible. This affects how well DL models perform. This study can be enhanced further by adding more photographs to the collection. In addition, as the proposed model extracts more detailed, accurate, and discriminating characteristics, testing with the dataset indicates that there are relatively few photos of cancer SC and a substantial number of photographs of normal skin. This contrasts with the fact that there are many images of normal skin. Before dividing the pictures obtained from a dermatoscopy of the skin into cancerous and non-cancerous types, the data on skin cancer should first be subjected to a successful segmentation method. After the photos have been segmented, the algorithm that has been suggested may utilize them to detect and distinguish malignant and non-cancerous images reliably. The direction of future studies may focus on answering therapeutically relevant questions. In addition, shortly, we want to investigate models such as ViT for use in SC detection. The successful use of enhanced DL algorithms may serve radiologists and oncologists in correctly identifying SC using MRI, dermatoscopy, and CT images. However, the results that were published in this research may help experts make the best selection of their models, eliminating the need for an exhaustive search. Utilizing deep neural networks solves many of the issues associated with model training. It enables us to develop accurate models for SC diagnosis, which aids in the early identification and treatment of the condition.

## Figures and Tables

**Table 1:** Attributes of the suggested SkinNetX Model.

Layer Type	Number of Filters
MobileNetV2	
GlobalAveragePooling2D	
Dense	256
Dropout	0.5
Dense	512
Xception	
GlobalAveragePooling2D	
Dense	256
Dropout	0.5
Dense	512
Concatenate	
Dense	2

**Table 2:** Pre-trained cutting-edge CNN models’ layers, parameters, and size.

Model	Depth	Parameters (M)	Size (MB)
AlexNet	11	60	227
DenseNet121	242	8.1	33
MobileNetV2	105	3.5	14
Xception	81	22.9	88
InceptionV3	189	23.9	92
ResNet50V2	103	25.6	98

**Table 3:** Parameters Used for Model Training.

Parameter	Values
Learning rate	0.0001
Optimizer	SGD
Epochs	100
Verbose	1
Activation function	ReLU
Iteration per epoch	12
Early stopping	Patience = 80

**Table 4:** Pre-trained Models Experimental Results.

Model	Accuracy	Precision	Recall	F1-Score	Misclass	AUC
Proposed Model	97.56	93.33	100	96.55	0.0244	97.00
Xception	85.37	93.33	73.68	82.35	0.1463	96.00
MobileNetV2	90.24	93.33	82.35	87.50	0.0976	95.00
AlexNet	80.49	80.00	70.59	75.00	0.1951	92.00
InceptionV3	82.93	70.00	93.33	80.00	0.1707	91.00
DenseNet121	95.12	93.33	93.33	93.33	0.0488	95.00
ResNet50V2	77.50	92.86	61.90	74.29	0.2250	86.00

**Table 5:** Comparative Evaluation with Existing Systems.

Authors	Models	Accuracy
Tembhurne et al. [96]	Cnoyrlet transform with LBP histograms and VGG19	93%
Mehr and Ameri [97]	Inception-ResNet-v2	94.5%
Huang et al. [98]	DenseNet121	89.5%
Proposed SkinNetX Model	Concatenation of MobileNet and Xception Models	97.56%

## Data Availability

The datasets employed in this study are obtainable on Kaggle: https://www.kaggle.com/datasets/kylegraupe/skin-cancer-binary-classification-dataset (retrieved on 16 June 2023).

## References

[1] Rembielak A., Ajithkumar T. (2019). Non-Melanoma Skin Cancer—An Underestimated Global Health Threat?. Clin. Oncol..

[2] Hopkins Z.H., Secrest A.M. (2019). Public health implications of Google searches for sunscreen, sunburn, skin cancer, and melanoma in the United States. Am. J. Health Promot..

[3] Santos M.O. (2018). Estimate 2018: Cancer incidence in Brazil. Rev. Bras. Cancerol..

[4] Adegun A.A., Viriri S., Ogundokun R.O. (2021). Deep Learning Approach for Medical Image Analysis. Comput. Intell. Neurosci..

[5] Adegun A.A., Ogundokun R.O., Adebiyi M.O., Asani E.O. (2020). CAD-based machine learning project for reducing human-factor-related errors in medical image analysis. Handbook of Research on the Role of Human Factors in IT Project Management.

[6] Ayo F.E., Ogundokun R.O., Awotunde J.B., Adebiyi M.O., Adeniyi A.E. (2020). Severe acne skin disease: A fuzzy-based method for diagnosis. Proceedings of the Computational Science and Its Applications–ICCSA 2020: 20th International Conference.

[7] Li Q.-K., Lin H., Tan X., Du S. (2020). H∞ Consensus for Multiagent-Based Supply Chain Systems Under Switching Topology and Uncertain Demands. IEEE Trans. Syst. Man Cybern. Syst..

[8] Tan X., Lin J., Xu K., Chen P., Ma L., Lau R.W. (2023). Mirror Detection with the Visual Chirality Cue. IEEE Trans. Pattern Anal. Mach. Intell..

[9] Zhang X., Lu Z., Yuan X., Wang Y., Shen X. (2021). L2-Gain Adaptive Robust Control for Hybrid Energy Storage System in Electric Vehicles. IEEE Trans. Power Electron..

[10] Liu N., Liang G., Li L., Zhou H., Zhang L., Song X. (2021). An eyelid parameters auto-measuring method based on 3D scanning. Displays.

[11] Raza K., Singh N.K. (2021). A tour of unsupervised deep learning for medical image analysis. Curr. Med. Imaging.

[12] Cheng L., Yin F., Theodoridis S., Chatzis S., Chang T.-H. (2022). Rethinking Bayesian Learning for Data Analysis: The art of prior and inference in sparsity-aware modeling. IEEE Signal Process. Mag..

[13] Li C., Lin L., Zhang L., Xu R., Chen X., Ji J., Li Y. (2021). Long noncoding RNA p21 enhances autophagy to alleviate endothelial progenitor cells damage and promote endothelial repair in hypertension through SESN2/AMPK/TSC2 pathway. Pharmacol. Res..

[14] Jin K., Gao Z., Jiang X., Wang Y., Ma X., Li Y., Ye J. (2023). MSHF: A Multi-Source Heterogeneous Fundus (MSHF) Dataset for Image Quality Assessment. Sci. Data.

[15] Xiong S., Li B., Zhu S. (2022). DCGNN: A single-stage 3D object detection network based on density clustering and graph neural network. Complex Intell. Syst..

[16] Deng X., Liu E., Li S., Duan Y., Xu M. (2023). Interpretable Multi-Modal Image Registration Network Based on Disentangled Convolutional Sparse Coding. IEEE Trans. Image Process..

[17] Dang W., Xiang L., Liu S., Yang B., Liu M., Yin Z., Yin L., Zheng W. (2023). A Feature Matching Method based on the Convolutional Neural Network. J. Imaging Sci. Technol..

[18] Lu S., Yang B., Xiao Y., Liu S., Liu M., Yin L., Zheng W. (2023). Iterative reconstruction of low-dose CT based on differential sparse. Biomed. Signal Process. Control..

[19] Xu H., Van der Jeught K., Zhou Z., Zhang L., Yu T., Sun Y., Li Y., Wan C., So K.M., Liu D. (2021). Atractylenolide I enhances responsiveness to immune checkpoint blockade therapy by activating tumor antigen presentation. J. Clin. Investig..

[20] Brinker T.J., Hekler A., Utikal J.S., Grabe N., Schadendorf D., Klode J., Berking C., Steeb T., Enk A.H., von Kalle C. (2018). Skin cancer classification using convolutional neural networks: Systematic review. J. Med. Internet Res..

[21] Lv Z., Qiao L., Li J., Song H. (2020). Deep-Learning-Enabled Security Issues in the Internet of Things. IEEE Internet Things J..

[22] Pan S.J., Yang Q. (2010). A survey on transfer learning. IEEE Trans. Knowl. Data Eng..

[23] Shorten C., Khoshgoftaar T.M. (2019). A survey on Image Data Augmentation for Deep Learning. J. Big Data.

[24] Nath R.P., Balaji V.N. (2014). Artificial intelligence in power systems. IOSR Journal of Computer Engineering (IOSR-JCE).

[25] Rashid J., Ishfaq M., Ali G., Saeed M.R., Hussain M., Alkhalifah T., Alturise F., Samand N. (2022). Skin Cancer Disease Detection Using Transfer Learning Technique. Appl. Sci..

[26] Xu Y., Ahokangas P., Louis J.N., Pongrácz E. (2019). Electricity market empowered by artificial intelligence: A platform approach. Energies.

[27] Kourou K., Exarchos T.P., Exarchos K.P., Karamouzis M.V., Fotiadis D.I. (2015). Machine learning applications in cancer prognosis and prediction. Comput. Struct. Biotechnol. J..

[28] Cruz J.A., Wishart D.S. (2006). Applications of Machine Learning in Cancer Prediction and Prognosis. Cancer Informatics.

[29] Sohail M., Ali G., Rashid J., Ahmad I., Almotiri S.H., AlGhamdi M.A., Nagra A.A., Masood K. (2021). Racial Identity-Aware Facial Expression Recognition Using Deep Convolutional Neural Networks. Appl. Sci..

[30] Rashid J., Khan I., Ali G., Almotiri S.H., AlGhamdi M.A., Masood K. (2021). Multi-Level Deep Learning Model for Potato Leaf Disease Recognition. Electronics.

[31] Arowolo M.O., Ogundokun R.O., Misra S., Agboola B.D., Gupta B. (2023). Machine learning-based IoT system for COVID-19 epidemics. Computing.

[32] Hordri N.F., Yuhaniz S.S., Shamsuddin S.M. Deep learning and its applications: A review. Proceedings of the Conference on Postgraduate Annual Research on Informatics Seminar.

[33] Zeng Q., Bie B., Guo Q., Yuan Y., Han Q., Han X., Chen M., Zhang X., Yang Y., Liu M. (2020). Hyperpolarized Xe NMR signal advancement by metal-organic framework entrapment in aqueous solution. Proc. Natl. Acad. Sci. USA.

[34] LeCun Y., Bengio Y., Hinton G. (2015). Deep learning. Nature.

[35] Omotosho A., Asani E.O., Ogundokun R.O., Ananti E.C., Adegun A. (2018). A neuro-fuzzy based system for the classification of cells as cancerous or non-cancerous. Int. J. Med. Res. Health Sci..

[36] Fujisawa Y., Inoue S., Nakamura Y. (2019). The possibility of deep learning-based, computer-aided skin tumor classifiers. Front. Med..

[37] Feng X., Yao H., Zhang S. (2019). An efficient way to refine DenseNet. Signal Image Video Process..

[38] Shin H.-C., Roth H.R., Gao M., Lu L., Xu Z., Nogues I., Yao J., Mollura D., Summers R.M. (2016). Deep convolutional neural networks for computer-aided detection: CNN architectures, dataset characteristics and transfer learning. IEEE Trans. Med. Imaging.

[39] Pham B.T., Jaafari A., Prakash I., Bui D.T. (2019). A novel hybrid intelligent model of support vector machines and the MultiBoost ensemble for landslide susceptibility modeling. Bull. Eng. Geol. Environ..

[40] Salamon J., Bello J.P. (2017). Deep Convolutional Neural Networks and Data Augmentation for Environmental Sound Classification. IEEE Signal Process. Lett..

[41] Matsunaga K., Hamada A., Minagawa A., Koga H. (2017). Image classification of melanoma, nevus and seborrheic keratosis by deep neural network ensemble. arXiv.

[42] Albahar M.A. (2019). Skin Lesion Classification Using Convolutional Neural Network With Novel Regularizer. IEEE Access.

[43] Ao J., Shao X., Liu Z., Liu Q., Xia J., Shi Y., Qi L., Pan J., Ji M. (2023). Stimulated Raman Scattering Microscopy Enables Gleason Scoring of Prostate Core Needle Biopsy by a Convolutional Neural Network. Cancer Res..

[44] Zhuang Y., Chen S., Jiang N., Hu H. (2022). An Effective WSSENet-Based Similarity Retrieval Method of Large Lung CT Image Databases. KSII Trans. Internet Inf. Syst..

[45] Zhuang Y., Jiang N., Xu Y. (2022). Progressive Distributed and Parallel Similarity Retrieval of Large CT Image Sequences in Mobile Telemedicine Networks. Wirel. Commun. Mob. Comput..

[46] Lu S., Yang J., Yang B., Yin Z., Liu M., Yin L., Zheng W. (2023). Analysis and Design of Surgical Instrument Localization Algorithm. Comput. Model. Eng. Sci..

[47] Wang Q., Sun L., Wang Y., Zhou M., Hu M., Chen J., Wen Y., Li Q. (2020). Identification of melanoma from hyperspectral pathology image using 3D convolutional networks. IEEE Trans. Med. Imaging.

[48] Tan S., Li D., Zhu X. (2020). Cancer immunotherapy: Pros, cons and beyond. Biomed. Pharmacother..

[49] Sinikumpu S.-P., Jokelainen J., Keinänen-Kiukaanniemi S., Huilaja L. (2022). Skin cancers and their risk factors in older persons: A population-based study. BMC Geriatr..

[50] Jinnai S., Yamazaki N., Hirano Y., Sugawara Y., Ohe Y., Hamamoto R. (2020). The Development of a Skin Cancer Classification System for Pigmented Skin Lesions Using Deep Learning. Biomolecules.

[51] Parker E.R. (2020). The influence of climate change on skin cancer incidence—A review of the evidence. Int. J. Women’s Dermatol..

[52] Efimenko M., Ignatev A., Koshechkin K. (2020). Review of medical image recognition technologies to detect melanomas using neural networks. BMC Bioinform..

[53] Zhuo Z., Du L., Lu X., Chen J., Cao Z. (2022). Smoothed Lv Distribution Based Three-Dimensional Imaging for Spinning Space Debris. IEEE Trans. Geosci. Remote. Sens..

[54] Ojukwu C.E. (2021). Melanoma skin cancer detection using support vector machines and convolutional neural networks. Int. J. Sci. Res. Comput. Sci. Eng..

[55] Mohapatra S., Abhishek NV S., Bardhan D., Ghosh A.A., Mohanty S. (2020). Skin cancer classification using convolution neural networks. Advances in Distributed Computing and Machine Learning: Proceedings of ICADCML 2020.

[56] Azghadi M.R., Lammie C., Eshraghian J.K., Payvand M., Donati E., Linares-Barranco B., Indiveri G. (2020). Hardware implementation of deep network accelerators towards healthcare and biomedical applications. IEEE Trans. Biomed. Circuits Syst..

[57] Zhang J., Shen Q., Ma Y., Liu L., Jia W., Chen L., Xie J. (2022). Calcium Homeostasis in Parkinson’s Disease: From Pathology to Treatment. Neurosci. Bull..

[58] Wang S., Hu X., Sun J., Liu J. (2023). Hyperspectral anomaly detection using ensemble and robust collaborative representation. Inf. Sci..

[59] Nie W., Bao Y., Zhao Y., Liu A. (2023). Long Dialogue Emotion Detection Based on Commonsense Knowledge Graph Guidance. IEEE Trans. Multimed..

[60] Gao Z., Pan X., Shao J., Jiang X., Su Z., Jin K., Ye J. (2022). Automatic interpretation and clinical evaluation for fundus fluorescein angiography images of diabetic retinopathy patients by deep learning. Br. J. Ophthalmol..

[61] Giotis I., Molders N., Land S., Biehl M., Jonkman M.F., Petkov N. (2015). MED-NODE: A computer-assisted melanoma diagnosis system using non-dermoscopic images. Expert Syst. Appl..

[62] Wang Y., Xu N., Liu A.-A., Li W., Zhang Y. (2022). High-Order Interaction Learning for Image Captioning. IEEE Trans. Circuits Syst. Video Technol..

[63] Yang S., Li Q., Li W., Li X., Liu A.-A. (2022). Dual-Level Representation Enhancement on Characteristic and Context for Image-Text Retrieval. IEEE Trans. Circuits Syst. Video Technol..

[64] Wang W., Chen Z., Yuan X. (2022). Simple low-light image enhancement based on Weber–Fechner law in logarithmic space. Signal Process. Image Commun..

[65] Zhao L., Wang L. (2022). A new lightweight network based on MobileNetV3. KSII Trans. Internet Inf. Syst..

[66] Khan M.Q., Hussain A., Rehman S.U., Khan U., Maqsood M., Mehmood K., Khan M.A. (2019). Classification of Melanoma and Nevus in Digital Images for Diagnosis of Skin Cancer. IEEE Access.

[67] Filali Y., ELKhoukhi H., Sabri M.A., Aarab A. (2020). Efficient fusion of hand-crafted and pre-trained CNNs features to classify melanoma skin cancer. Multimed. Tools Appl..

[68] Hu K., Niu X., Liu S., Zhang Y., Cao C., Xiao F., Yang W., Gao X. (2019). Classification of melanoma based on feature similarity measurement for codebook learning in the bag-of-features model. Biomed. Signal Process. Control..

[69] Abbas Q., Celebi M.E. (2019). DermoDeep-A classification of melanoma-nevus skin lesions using multi-feature fusion of visual features and deep neural network. Multimed. Tools Appl..

[70] Dalila F., Zohra A., Reda K., Hocine C. (2017). Segmentation and classification of melanoma and benign skin lesions. Optik.

[71] Almansour E., Jaffar M.A. (2016). Classification of Dermoscopic skin cancer images using color and hybrid texture features. IJCSNS.

[72] Pham T.C., Luong C.M., Visani M., Hoang V.D. (2018). Deep CNN and data augmentation for skin lesion classification. Proceedings of the Intelligent Information and Database Systems: 10th Asian Conference, ACIIDS 2018.

[73] Yu L., Chen H., Dou Q., Qin J., Heng P.A. (2016). Automated Melanoma Recognition in Dermoscopy Images via Very Deep Residual Networks. IEEE Trans. Med. Imaging.

[74] Rokhana R., Herulambang W., Indraswari R. Deep convolutional neural network for melanoma image classification. Proceedings of the 2020 International Electronics Symposium (IES).

[75] Xie F.-Y., Fan H., Li Y., Jiang Z.-G., Meng R.-S., Bovik A. (2016). Melanoma Classification on Dermoscopy Images Using a Neural Network Ensemble Model. IEEE Trans. Med. Imaging.

[76] Liberman G., Acevedo D., Mejail M. (2018). Classification of melanoma images with fisher vectors and deep learning. Iberoamerican Congress on Pattern Recognition.

[77] Zhou Q., Shi Y., Xu Z., Qu R., Xu G. (2020). Classifying melanoma skin lesions using convolutional spiking neural networks with unsupervised stdp learning rule. IEEE Access.

[78] Hosny K.M., Kassem M.A., Foaud M.M. (2020). Skin melanoma classification using ROI and data augmentation with deep convolutional neural networks. Multimed. Tools Appl..

[79] Mukherjee S., Adhikari A., Roy M. (2019). Malignant melanoma classification using cross-platform dataset with deep learning CNN architecture. Recent Trends in Signal and Image Processing.

[80] Esteva A., Kuprel B., Thrun S. (2015). Deep Networks for Early Stage Skin Disease and Skin Cancer Classification.

[81] Çakmak M., Tenekecı M.E. Melanoma detection from dermoscopy images using Nasnet Mobile with Transfer Learning. Proceedings of the 2021 29th Signal Processing and Communications Applications Conference (SIU).

[82] Han S.S., Kim M.S., Lim W., Park G.H., Park I., Chang S.E. (2018). Classification of the clinical images for benign and malignant cutaneous tumors using a deep learning algorithm. J. Investig. Dermatol..

[83] Hosny K.M., Kassem M.A., Foaud M.M. (2019). Classification of skin lesions using transfer learning and augmentation with Alex-net. PLoS ONE.

[84] Esteva A., Kuprel B., Novoa R.A., Ko J., Swetter S.M., Blau H.M., Thrun S. (2017). Dermatologist-level classification of skin cancer with deep neural networks. Nature.

[85] Ogundokun R.O., Misra S., Douglas M., Damaševičius R., Maskeliūnas R. (2022). Medical Internet-of-Things Based Breast Cancer Diagnosis Using Hyperparameter-Optimized Neural Networks. Futur. Internet.

[86] Jordan M.I., Mitchell T.M. (2015). Machine learning: Trends, perspectives, and prospects. Science.

[87] Huang G., Liu Z., Van Der Maaten L., Weinberger K.Q. Densely connected convolutional networks. Proceedings of the IEEE Conference on Computer Vision and Pattern Recognition.

[88] Ogundokun R.O., Maskeliūnas R., Damaševičius R. (2022). Human posture detection using image augmentation and hyperparameter-optimized transfer learning algorithms. Appl. Sci..

[89] Sharma S., Kumar S. (2022). The Xception model: A potential feature extractor in breast cancer histology images classification. ICT Express.

[90] Chollet F. Xception: Deep learning with depthwise separable convolutions. Proceedings of the IEEE Conference on Computer Vision and Pattern Recognition.

[91] Dong K., Zhou C., Ruan Y., Li Y. MobileNetV2 model for image classification. Proceedings of the 2020 2nd International Conference on Information Technology and Computer Application (ITCA).

[92] Ahsan M., Nazim R., Siddique Z., Huebner P. (2021). Detection of COVID-19 Patients from CT Scan and Chest X-ray Data Using Modified *MobileNetV2* and *LIME*. Healthcare.

[93] Xiao L., Yan Q., Deng S. Scene classification with improved AlexNet model. Proceedings of the 2017 12th International Conference on Intelligent Systems and Knowledge Engineering (ISKE).

[94] Nandhini S., Ashokkumar K. (2022). An automatic plant leaf disease identification using DenseNet-121 architecture with a mutation-based henry gas solubility optimization algorithm. Neural Comput. Appl..

[95] Zhou Z., Yang X., Ji J., Wang Y., Zhu Z. (2023). Classifying fabric defects with evolving Inception v3 by improved L2, 1-norm regularized extreme learning machine. Text. Res. J..

[96] Tembhurne J.V., Hebbar N., Patil H.Y., Diwan T. (2023). Skin cancer detection using ensemble of machine learning and deep learning techniques. Multimed. Tools Appl..

[97] Mehr R.A., Ameri A. (2022). Skin Cancer Detection Based on Deep Learning. J. Biomed. Phys. Eng..

[98] Huang H., Hsu B.W., Lee C., Tseng V.S. (2021). Development of a light-weight deep learning model for cloud applications and remote diagnosis of skin cancers. J. Dermatol..

[99] Chollet F. (2015). GitHub—Keras-Team/Keras: Deep Learning for Humans. https://github.com/keras-team/keras.

[100] (2019). FAQ—Keras Documentation. https://keras.io/getting-started/faq/#why-is-the-training-loss-much-higher-than-the-testing-loss.

